# Supplementary material for: A molecular pyroelectric enabling broadband photo-pyroelectric effect towards self-driven wide spectral photodetection
Source: Nat Commun. 2023 Sep 19;14:5821. doi: 10.1038/s41467-023-41523-z (PMC10509268; doi:10.1038/s41467-023-41523-z)
Supplement: Supplementary file 1 — Supplementary information [file 41467_2023_41523_MOESM1_ESM.pdf]

# Supplementary information

# A molecular pyroelectric enabling broadband photo-pyroelectric effect towards self-driven wide spectral photodetection

Xi Zeng[1,2], Yi Liu[1,2], Wen Weng[1,2], Lina Hua[1], Liwei Tang[1], Wuqian Guo[1,2], Yaoyao Chen[1], Tian Yang[1], Haojie Xu[1,2], Junhua Luo[1,2,3,*] and Zhihua Sun[1,2,3,*]

[1]State Key Laboratory of Structural Chemistry, Fujian Institute of Research on the Structure of Matter, Chinese Academy of Sciences, Fuzhou, Fujian 350002, China

[2]University of Chinese Academy of Sciences, Beijing 100049, China

[3]Fujian Science & Technology Innovation Laboratory for Optoelectronic Information of China, Fuzhou, Fujian 350108, China

***Corresponding authors:** jhluo@fjirsm.ac.cn; sunzhihua@fjirsm.ac.cn

## Supplementary Figures

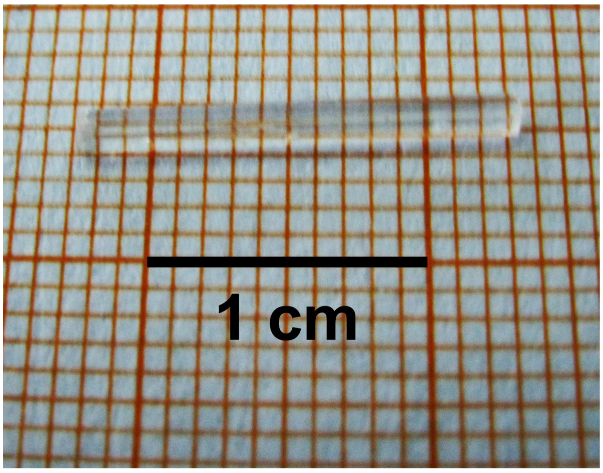


**Supplementary Fig. 1.** Colorless large-size crystal of *N*-IBATFA.

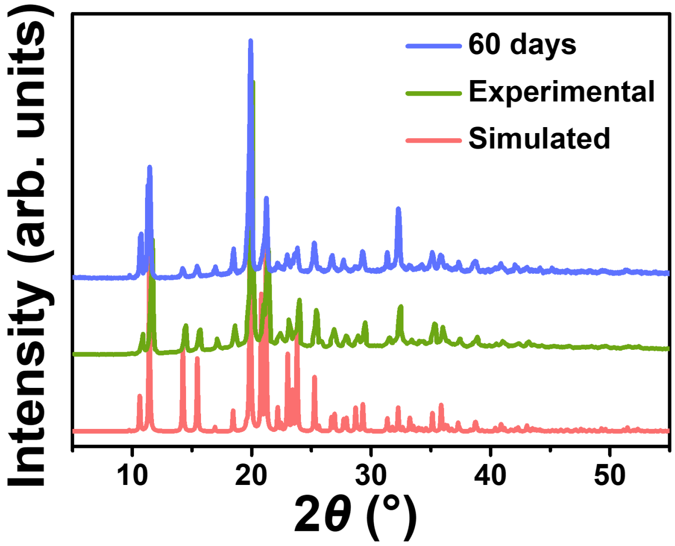


**Supplementary Fig. 2.** Simulated and experimental (fresh and after 60 days) PXRD patterns of *N*-IBATFA at room temperature.

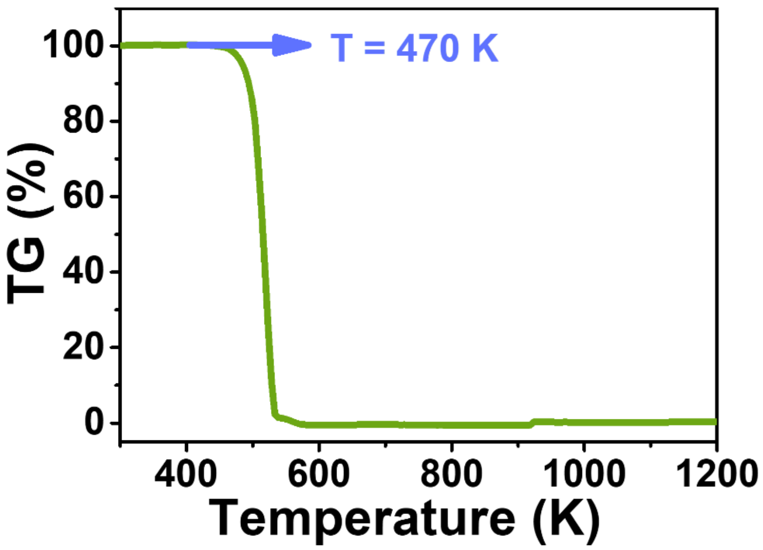


**Supplementary Fig. 3.** Analysis on the thermal stability of *N*-IBATFA. The result indicates that *N*-IBATFA possesses thermal stability up to ~470 K.

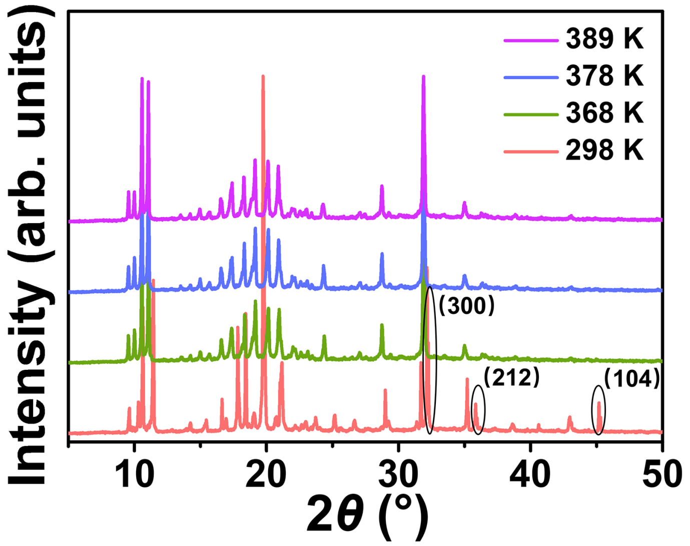


**Supplementary Fig. 4.** PXRD patterns of *N*-IBATFA in the temperature range 298-389 K. The occurrence of structural phase transition is clearly shown below and above the $T_c$. During the warming process from 298 K to $T_c$, the main Bragg diffraction peaks remain unchanged, while some diffractions of (300), (212) and (104) disappear, verifying the phase transition.

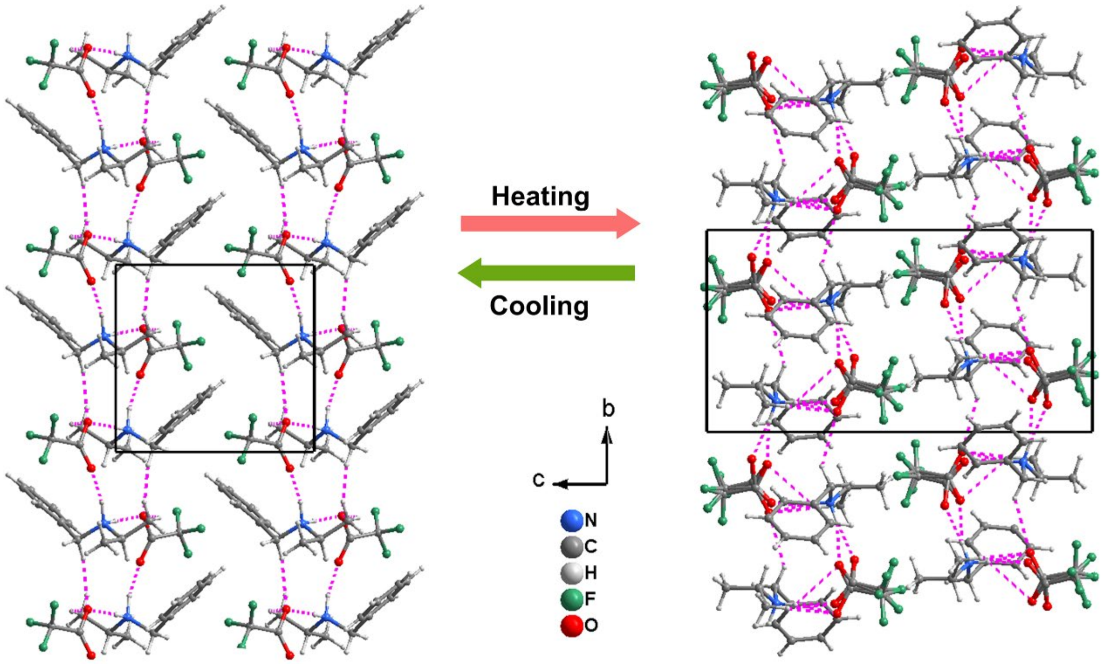


**Supplementary Fig. 5.** Packing diagram of *N*-IBATFA at LTP (left) and HTP (right) along the *a*-axis (H-bonds are represented by the magenta dotted lines).

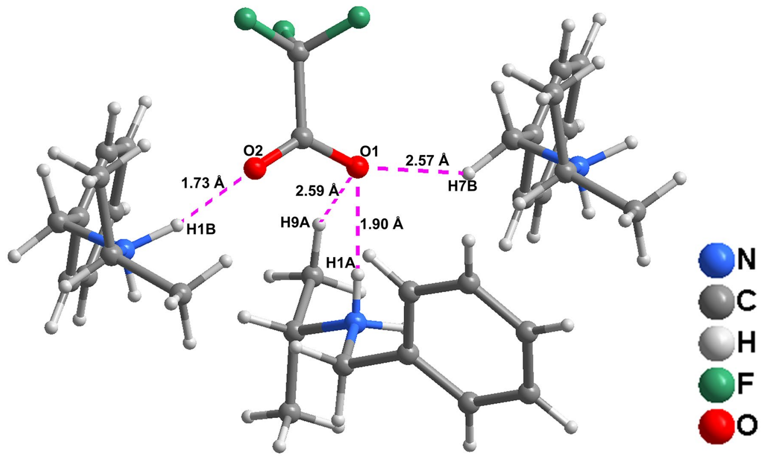


**Supplementary Fig. 6.** N-H···O hydrogen bonds of *N*-IBATFA at LTP.

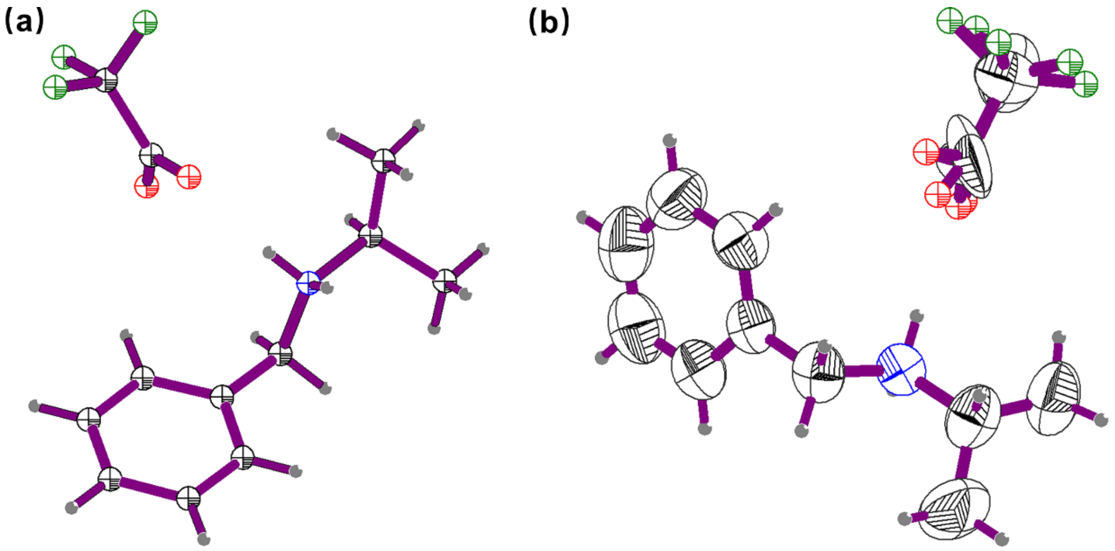


**Supplementary Fig. 7**. The thermal ellipsoid variation of *N*-IBATFA at LTP (a) and HTP (b), respectively.

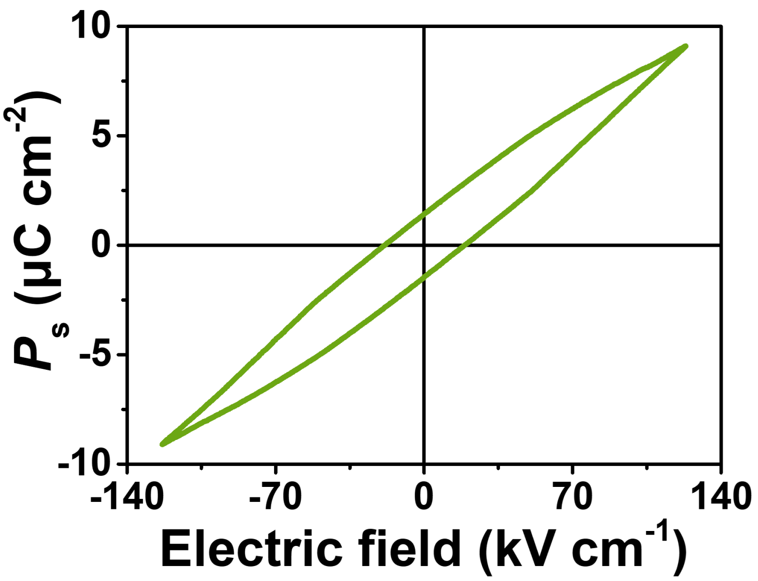


**Supplementary Fig. 8**. The *P*-*E* hysteresis loop measured along the polar axis of *N*-IBATFA crystal at LTP.

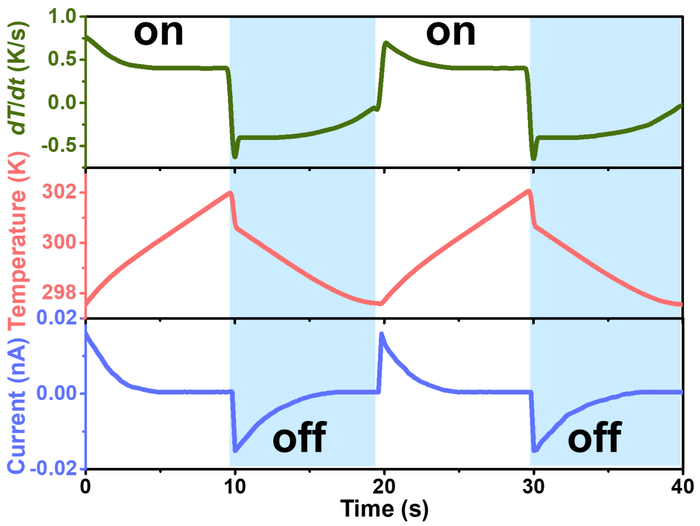


**Supplementary Fig. 9.** The photoexcited pyroelectric current response, cyclic change in temperature, and the corresponding differential curve of *N*-IBATFA under a periodically switched 405 nm laser with power density of 160 mW $cm^{-2}$.

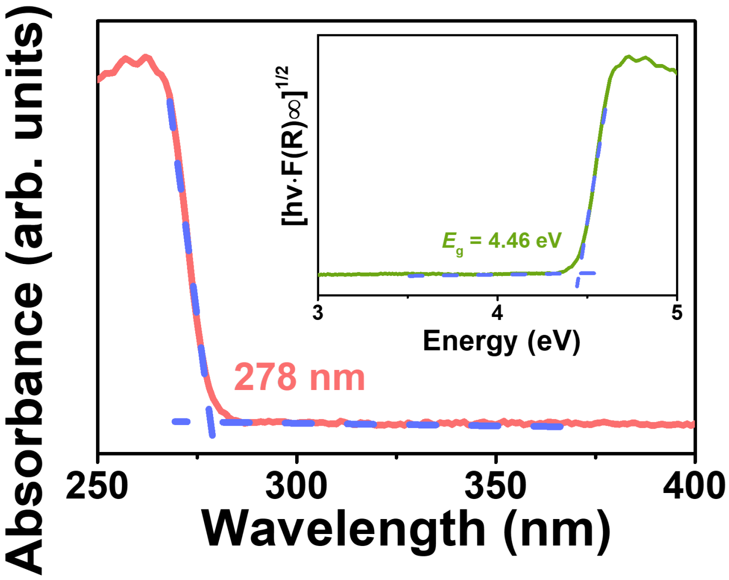


**Supplementary Fig. 10.** UV-vis spectra of *N*-IBATFA. Inset: the optical bandgap.

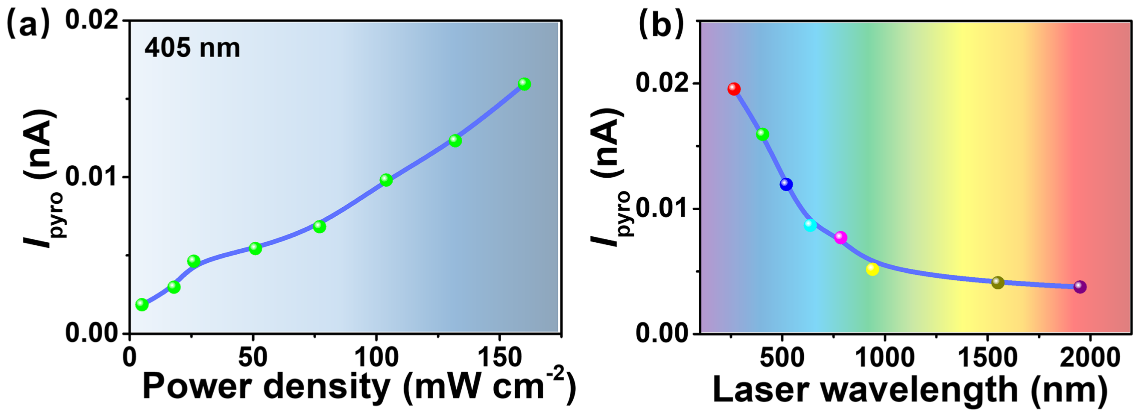


**Supplementary Fig. 11.** (a) The photoexcited pyroelectric current peak as a function of power density under 405 nm laser irradiation, *i.e.*, the current peak increases with increasing power density. (b) The photoexcited pyroelectric current peak as a function of incident wavelength with power density of 160 mW cm$^{-2}$, *i.e.*, the current peak decreases with the increasing of incident wavelength.

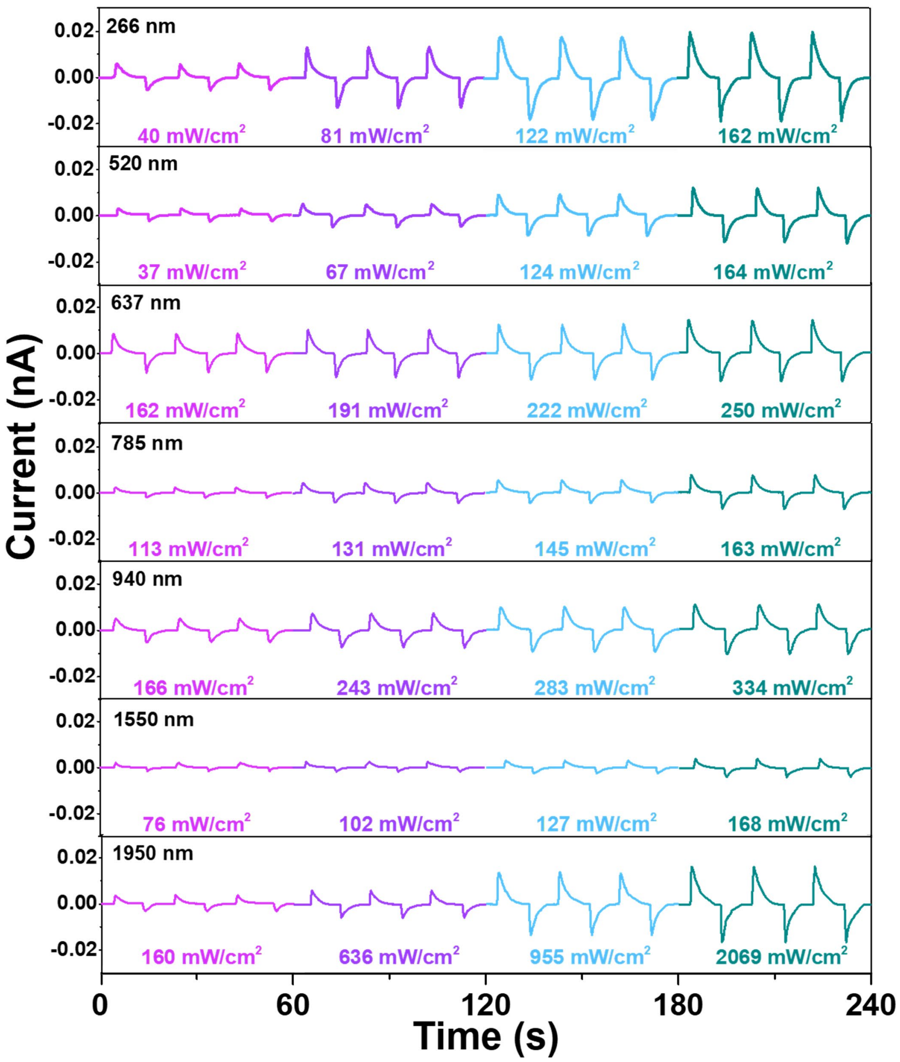


**Supplementary Fig. 12.** Broadband photoresponse behaviors under 266, 520, 637, 785, 949, 1550 and 1950 nm laser illuminations at 0 V bias voltage.

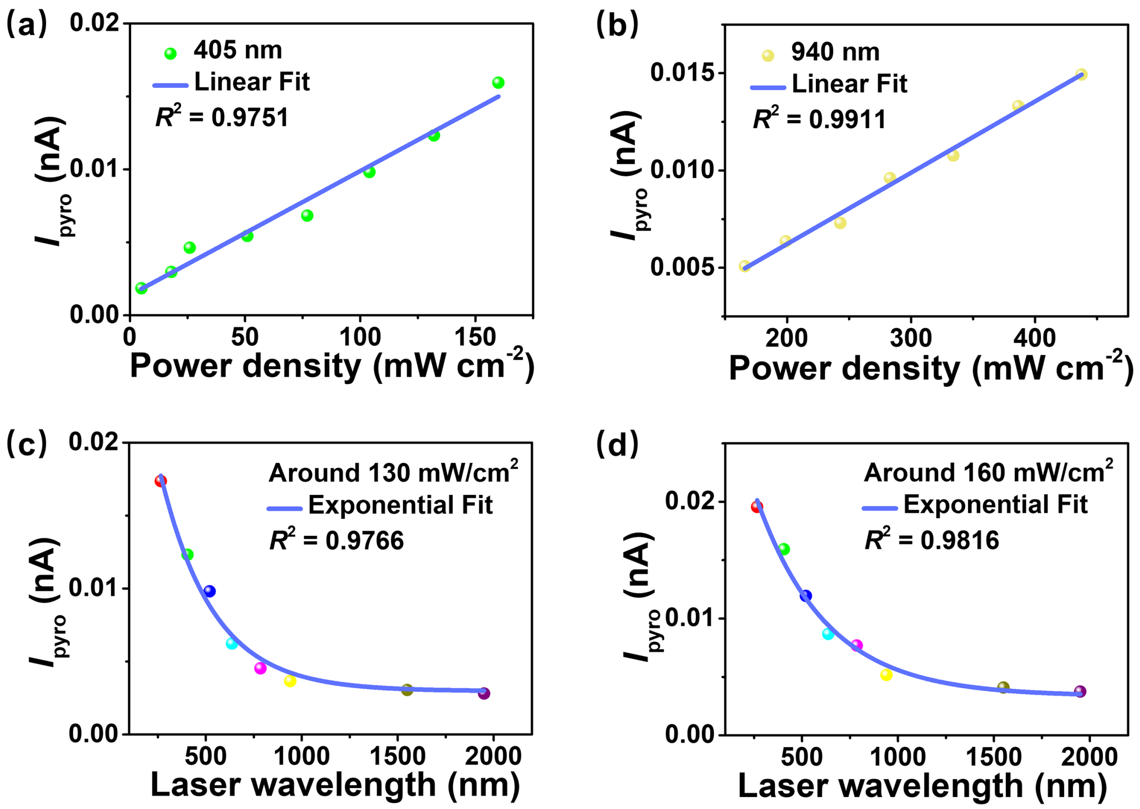


**Supplementary Fig. 13.** The photoexcited pyroelectric current peak as a function of power density under 405 nm (a) and 940 nm (b) laser irradiation. The photoexcited pyroelectric current peak as an exponential function of incident wavelength with a power density of around 130 mW cm$^{-2}$ (c) and 160 mW cm$^{-2}$ (d). At the fixed wavelengths such as 405 nm or 520 nm laser irradiation, an almost linear relationship exists between $I_{pyro}$ and power density. Consequently, the power density of a specific laser wavelength might be inferred from the observed $I_{pyro}$. And at fixed power density such as 130 mW cm$^{-2}$ or 160 mW cm$^{-2}$, there is an exponential relationship between $I_{pyro}$ and wavelength under laser irradiation at the selected wavelengths. Hence, the wavelength of a particular laser could be deduced by analyzing $I_{pyro}$ when irradiated at a fixed power density. These findings reveal that *N*-IBATFA might be a potential functional material capable of discerning the power density at a specific incident light wavelength and the incident light wavelength at a specific power density.

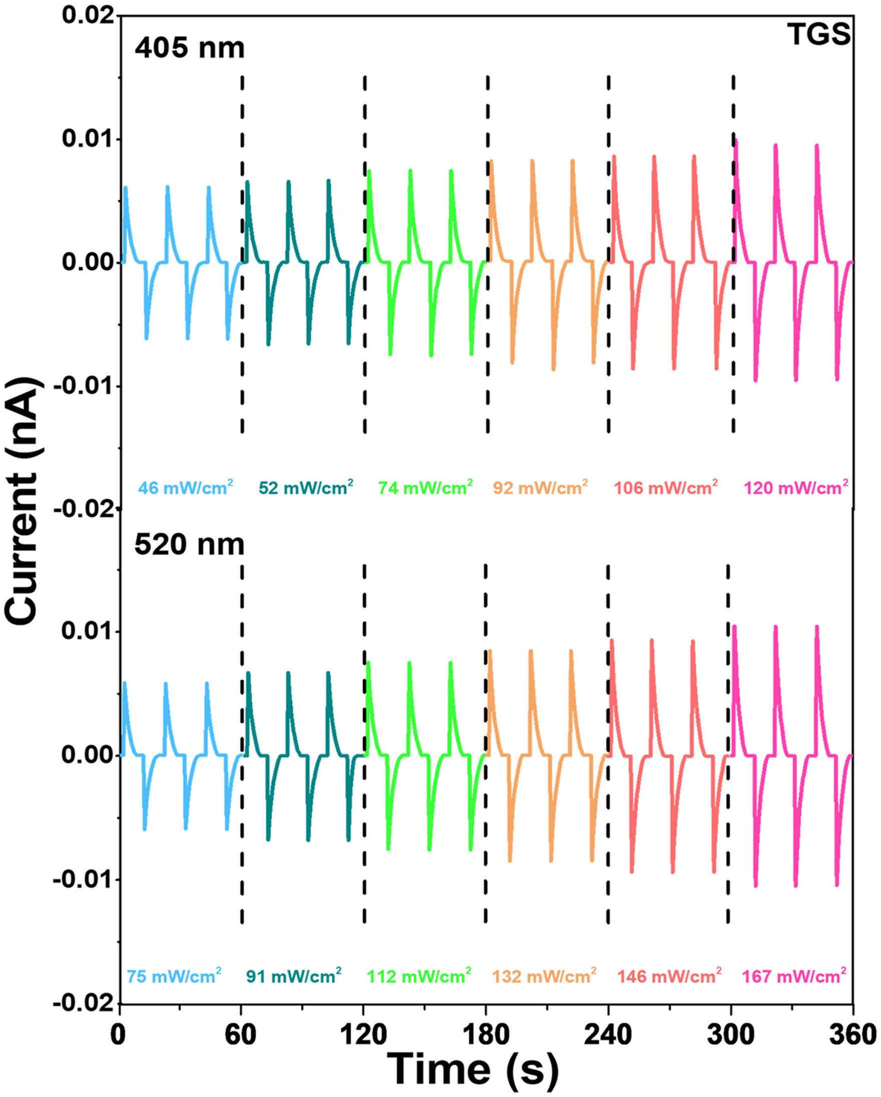


**Supplementary Fig. 14.** Variations of current intensity as a function of power density at 405 nm and 520 nm for TGS crystal.

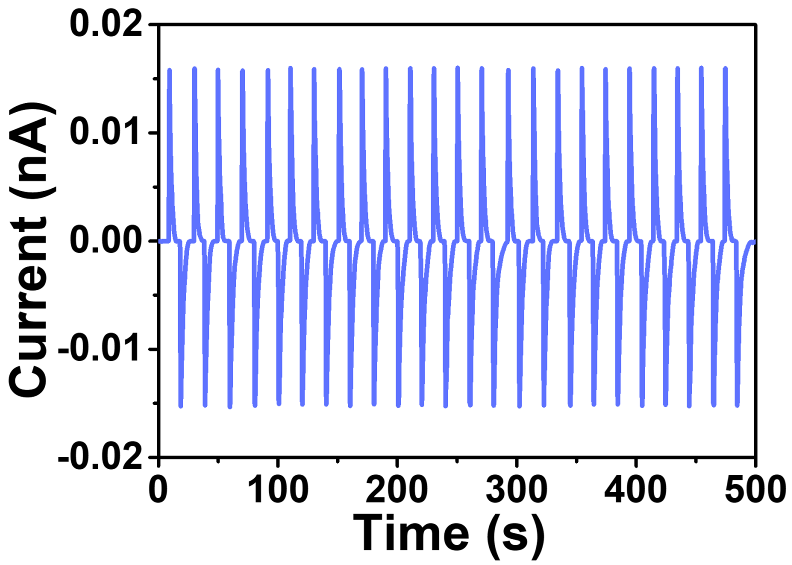


**Supplementary Fig. 15.** Repeated switching cycles of photoexcited pyroelectric current signals.

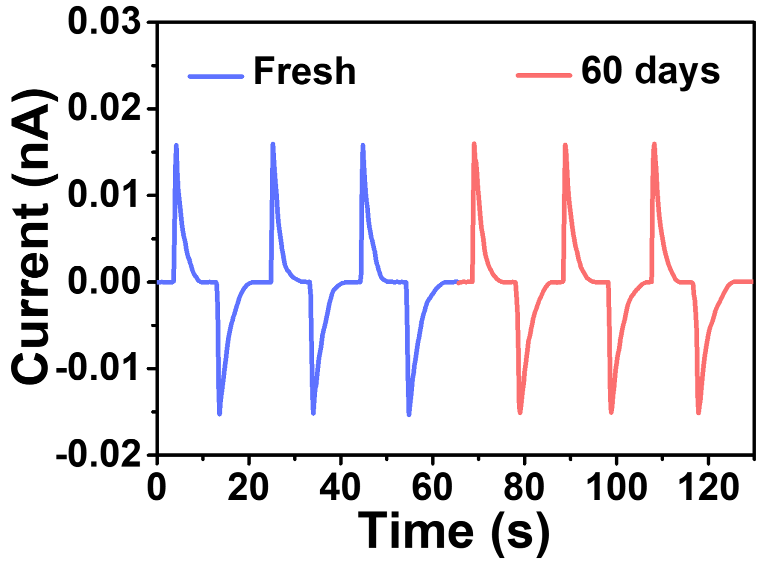


**Supplementary Fig. 16.** Photoexcited pyroelectric *I-t* curves of fresh sample and sample exposed to air for 60 days.

## Supplementary Tables

**Supplementary Table 1.** Crystal data for *N*-IBATFA collected at low-temperature phase (LTP, 293 K) and high-temperature phase (HTP, 365 K), respectively.

| Empirical formula | $C_{12}H_{16}F_3NO_2$ | $C_{12}H_{16}F_3NO_2$ |
|---|---|---|
| Formula weight | 263.26 | 263.26 |
| Temperature/K | 293 | 365 |
| Crystal system | monoclinic | monoclinic |
| Space group | $P2_1$ | $P2_1/n$ |
| $a$/Å | 8.965(6) | 9.7741(15) |
| $b$/Å | 8.533(5) | 8.7965(9) |
| $c$/Å | 9.779(7) | 16.959(2) |
| $\beta$/° | 111.918(9) | 96.341(12) |
| Volume/Å$^3$ | 694.0(8) | 1449.2(3) |
| Z | 2 | 4 |
| $\rho_{calc}$ g/cm$^3$ | 1.260 | 1.207 |
| $\mu$/mm$^{-1}$ | 0.112 | 0.107 |
| $F$(000) | 276 | 552 |
| Radiation | Mo$K\alpha$ (λ = 0.71073) | Mo$K\alpha$ (λ = 0.71073) |
| 2$\Theta$ range for data collection/° | 6.842 to 54.94 | 6.248 to 50.054 |
| Index ranges | $-11 \le h \le 11$, $-10 \le k \le 11$, $-12 \le l \le 12$ | $-11 \le h \le 11$, $-10 \le k \le 10$, $-20 \le l \le 19$ |
| Reflections collected | 5223 | 7510 |
| Independent reflections | 2935 [$R_{int}$ = 0.0121, $R_{sigma}$ = 0.0275] | 2546 [$R_{int}$ = 0.0899, $R_{sigma}$ = 0.0651] |
| Data/restraints/parameters | 2935/1/173 | 2546/127/235 |
| Goodness-of-fit on $F^2$ | 1.026 | 1.006 |
| Final $R$ indexes [$I$>=2$\sigma$ (I)] | $R_1$ = 0.0821, $wR_2$ = 0.2390 | $R_1$ = 0.0787, $wR_2$ = 0.1193 |
| Final $R$ indexes [all data] | $R_1$ = 0.0929, $wR_2$ = 0.2543 | $R_1$ = 0.1693, $wR_2$ = 0.1476 |

**Supplementary Table 2.** Hydrogen bonds of *N*-IBATFA at 293 and 365 K.

| | D-H···A | d(D-H) | d(H···A) | <DHA | d(D···A) |
|---|---|---|---|---|---|
| 293 K | N1-H1A···O1[1] | 0.86(6) | 1.90(7) | 175(6) | 2.763(5) |
| | N1-H1B···O2[2] | 1.03(6) | 1.73(6) | 167(5) | 2.743(6) |
| | C7-H7B···O1 | 0.97 | 2.57 | 130.8 | 3.292(7) |
| | C9-H9A···O1[1] | 0.96 | 2.59 | 136.4 | 3.353(8) |
| 365 K | N1-H1A···O1[1] | 0.87(4) | 2.59(4) | 135(3) | 3.270(12) |
| | N1-H1A···O2[1] | 0.87(4) | 2.01(4) | 170(4) | 2.873(14) |
| | N1-H1A···O2B[1] | 0.87(4) | 1.85(4) | 174(4) | 2.72(2) |
| | N1-H1B···O1 | 0.94(4) | 1.86(4) | 162(3) | 2.764(11) |
| | N1-H1B···O1B | 0.94(4) | 1.80(4) | 168(3) | 2.72(2) |
| | C7-H7B···O2[2] | 0.97 | 2.54 | 139.7 | 3.343(9) |
| | C10-H10A···O2[1] | 0.96 | 2.63 | 139.8 | 3.422(17) |
| | C10-H10A···O2B[1] | 0.96 | 2.61 | 135.5 | 3.37(3) |

Symmetry transformations used to generate equivalent atoms: [1]1-X,-1/2+Y,2-Z; [2]+X,-1+Y,+Z; [1]3/2-X,-1/2+Y,1/2-Z; [2]+X,-1+Y,+Z.

**Supplementary Table 3.** Selected bond angles of *N*-IBATFA at 293 and 365 K.

| | Atom | Atom | Atom | Angle/° | Atom | Atom | Atom | Angle/° |
|---|---|---|---|---|---|---|---|---|
| 293 K | C7 | N1 | C8 | 116.6(4) | C10 | C8 | N1 | 111.2(5) |
| | C2 | C1 | C7 | 121.5(5) | C10 | C8 | C9 | 111.8(5) |
| | C6 | C1 | C2 | 117.0(5) | O1 | C11 | C12 | 112.2(4) |
| | C6 | C1 | C7 | 121.4(5) | O2 | C11 | O1 | 129.1(5) |
| | C3 | C2 | C1 | 121.1(5) | O2 | C11 | C12 | 118.7(5) |
| | C4 | C3 | C2 | 120.9(7) | F1 | C12 | F3 | 103.0(7) |
| | C3 | C4 | C5 | 120.0(6) | F1 | C12 | C11 | 114.9(7) |
| | C6 | C5 | C4 | 119.2(6) | F2 | C12 | F1 | 100.1(9) |
| | C1 | C6 | C5 | 121.7(6) | F2 | C12 | F3 | 110.0(9) |
| | C1 | C7 | N1 | 111.6(4) | F2 | C12 | C11 | 114.8(4) |
| | N1 | C8 | C9 | 107.8(4) | F3 | C12 | C11 | 112.7(5) |
| 365 K | C7 | N1 | C8 | 116.0(3) | F1 | C12 | C11 | 112.0(15) |
| | C2 | C1 | C6 | 118.5(5) | F1 | C12 | F2 | 107.5(16) |
| | C2 | C1 | C7 | 119.9(5) | F1 | C12 | F3 | 110.0(15) |
| | C6 | C1 | C7 | 121.7(5) | F2 | C12 | C11 | 111.6(14) |
| | C1 | C2 | C3 | 120.3(6) | F2 | C12 | F3 | 107.4(16) |
| | C5 | C3 | C2 | 120.8(7) | F3 | C12 | C11 | 108.1(15) |
| | C5 | C4 | C6 | 119.1(6) | O1B | C11B | O2B | 125(3) |
| | C3 | C5 | C4 | 120.7(8) | O1B | C11B | C12B | 117(3) |
| | C1 | C6 | C4 | 120.7(5) | O2B | C11B | C12B | 110(3) |
| | N1 | C7 | C1 | 111.8(3) | F1B | C12B | F3B | 111(2) |
| | C9 | C8 | N1 | 109.6(5) | F1B | C12B | C11B | 101(3) |
| | C9 | C8 | C10 | 111.6(5) | F2B | C12B | F1B | 111(2) |
| | C10 | C8 | N1 | 107.8(4) | F2B | C12B | F3B | 113(2) |
| | O1 | C11 | O2 | 123.5(11) | F2B | C12B | C11B | 118(3) |
| | O1 | C11 | C12 | 122.3(12) | F3B | C12B | C11B | 102(2) |
| | O2 | C11 | C12 | 113.7(12) | | | | |

**Supplementary Table 4.** Selected bond lengths of crystal *N*-IBATFA at 293 and 365 K.

| | Atom | Atom | Length/Å | Atom | Atom | Length/Å |
|---|---|---|---|---|---|---|
| 293 K | N1 | C7 | 1.490(7) | C8 | C9 | 1.524(9) |
| | N1 | C8 | 1.504(5) | C8 | C10 | 1.498(9) |
| | C1 | C2 | 1.388(7) | F1 | C12 | 1.263(9) |
| | C1 | C6 | 1.379(7) | F2 | C12 | 1.204(7) |
| | C1 | C7 | 1.476(7) | F3 | C12 | 1.294(8) |
| | C2 | C3 | 1.366(8) | O1 | C11 | 1.254(6) |
| | C3 | C4 | 1.341(11) | O2 | C11 | 1.208(6) |
| | C4 | C5 | 1.380(11) | C12 | C11 | 1.522(7) |
| | C5 | C6 | 1.374(10) | | | |
| 365 K | N1 | C7 | 1.478(5) | O2 | C11 | 1.239(14) |
| | N1 | C8 | 1.513(5) | C11 | C12 | 1.511(13) |
| | C1 | C2 | 1.355(6) | C12 | F1 | 1.246(16) |
| | C1 | C6 | 1.355(5) | C12 | F2 | 1.258(16) |
| | C1 | C7 | 1.493(5) | C12 | F3 | 1.299(16) |
| | C2 | C3 | 1.380(7) | O1B | C11B | 1.198(18) |
| | C3 | C5 | 1.328(7) | O2B | C11B | 1.215(18) |
| | C4 | C5 | 1.336(7) | F1B | C12B | 1.26(2) |
| | C4 | C6 | 1.402(6) | F2B | C12B | 1.23(2) |
| | C8 | C9 | 1.474(7) | F3B | C12B | 1.26(2) |
| | C8 | C10 | 1.507(6) | C11B | C12B | 1.539(19) |
| | O1 | C11 | 1.201(11) | | | |

**Supplementary Table 5.** Pyroelectric properties of some typical pyroelectric materials.

| Materials | Curie temperature (K) | $P_e$ (μC cm$^{-2}$ K$^{-1}$) | $F_V$ ($10^{-2}$ cm$^2$ μC$^{-1}$) | $F_D$ ($10^{-5}$ Pa$^{-0.5}$) | Ref. |
|---|---|---|---|---|---|
| *N*-IBATFA | 360 | 6.9 | 187.9 | 881.5 | This work |
| TGS | 322 | 0.055 | 0.434 | 6.06 | 1, 2 |
| DTGS | 334 | 0.055 | 0.602 | 8.31 | 1, 3 |
| TGFB | 346 | 0.04 | 0.35 | 4.4 | 1, 4 |
| [Hdabco]$ClO_4$ | 373 | 4.8 | 5.58 | 25.2 | 5 |
| [Hdabco]$BF_4$ | 370 | 3.82 | 6.19 | 38.1 | 5 |
| Amantadine formate | 327 | 0.01 | 0.3 | 5.2 | 6 |
| Di-*n*-butylaminium trifluoroacetate | 212 | 0.55 | 0.58 | - | 7 |
| $LiTaO_3$ | 891 | 0.98 | - | - | 8 |
| $LiNbO_3$ | 1483 | 0.0096 | 0.14 | - | 9 |
| PMN-PT | 363 | 0.3 | 0.007 | 1.0 | 10 |
| $Sr_{0.5}Ba_{0.5}Nb_2O_6$ | 388 | 0.09 | 0.012 | 0.04 | 11 |
| $Ba_{0.85}Ca_{0.15}Zr_{0.1}Ti_{0.9}O_3$ | 322 | 0.098 | 0.009 | 0.79 | 12 |
| $(BA)_2(EA)_2Pb_3I_{10}$ | 315 | 1.1 | 30.04 | 612.0 | 13 |
| PVDF-TrFE | 333 | 0.0142 | 0.221 | 1.89 | 14, 15 |

**Supplementary Table 6.** Performance comparison with broadband photodetectors.

| Materials | Spectral region (nm) | Bias (V) | *R* (mA W$^{-1}$) | *D** ($10^{10}$ Jones) | Ref. |
|---|---|---|---|---|---|
| *N*-IBATFA | 266-1950 | 0 | 1.1 | 2.3 | This work |
| Multilayer $WS_2$ | 457-647 | 5 | 0.092 | - | 16 |
| Multilayer b-P | 640-940 | 0.2 | 4.8 | - | 17 |
| Single-layer $MoS_2$ | 400-1000 | 1 | 8 | - | 18 |
| $SnS_2$ nanosheet microsphere film | 300-830 | 5 | 0.206 | - | 19 |
| Metallic plasmon-graphene | 450-650 | 0 | 2.2 | - | 20 |
| ITO/SnS/Si/Al | 300-1100 | 0 | 8.3 | 0.53 | 21 |
| ITO/BTO/Ag | 365-690 | 0 | $3.3 \times 10^{-4}$ | $3.6 \times 10^{-5}$ | 22 |
| Au/PLZT/FTO | 250-600 | 0 | 0.011 | 0.044 | 23 |
| $WSe_2$/Si heterojunction | 365-940 | 3 | 2 | 0.024 | 24 |
| GQDs/$WSe_2$/Si heterojunction | 365-940 | 3 | 707 | 0.451 | 24 |
| Graphene/Si heterojunction | 400-900 | -2 | 225 | 0.021 | 25 |
| CuO/Si *p-n* junction | 405-1064 | 0 | 0.389 | 0.3 | 26 |
| $TiO_2$/NiO *p-n* junction | 280-600 | 0 | 0.042 | 0.11 | 27 |

## Supplementary References


1. Felix, P. et al. Pyroelectric, dielectric and thermal properties of TGS, DTGS and TGFB. *Ferroelectrics* **17**, 543-551 (1978).
2. Lock, P. J. Doped triglycine sulfate for pyroelectric applications. *Appl. Phys. Lett.* **19**, 390-391 (1971).
3. Bogomolov, A. A.; Malyshkina, O. V.; Solnyshkin, A. V. Pyroelectric effect in DTGS crystals under stationary temperature gradient. *J. Korean Phys. Soc.* **32**, S219-S220 (1998).
4. Bhalla, A. S. et al. Pyroelectric properties of the modified triglycine sulphate (TGS) single crystals. *Ferroelectrics* **54**, 491-494 (1984).
5. Li, W. et al. Improper molecular ferroelectrics with simultaneous ultrahigh pyroelectricity and figures of merit. *Sci. Adv.* **7**, eabe3068 (2021).
6. Zhou, J. et al. Discovery of amantadine formate: Toward achieving ultrahigh pyroelectric performances in organics. *The Innovation* **3**, 100204 (2022).
7. Sun, Z. et al. Ultrahigh pyroelectric figures of merit associated with distinct bistable dielectric phase transition in a new molecular compound: Di-*n*-butylaminium trifluoroacetate. *Adv. Mater.* **27**, 4795-4801 (2015).
8. Glass, A. M. Dielectric, thermal, and pyroelectric properties of ferroelectric $LiTaO_3$. *Phys. Rev.* **172**, 564-571 (1968).
9. Levy, M. et al. Fabrication of single-crystal lithium niobate films by crystal ion slicing. *Appl. Phys. Lett.* **73**, 2293-2295 (1998).
10. Park, J. H. et al. Electric-field induced strains and pyroelectric coefficients in lead magnesium niobate-lead titanate solid solutions. *Mater. Res. Bull.* **30**, 435-441 (1995).
11. Jimenez, B. et al. Phase transitions in ferroelectric ceramics of the type $Sr_{0.5}Ba_{0.5}Nb_2O_6$. *J. Phys. Chem. Solids* **46**, 1383-1386 (1985).
12. Patel, S.; Chauhan, A.; Vaish, R. Large pyroelectric figure of merits for Sr-modified $Ba_{0.85}Ca_{0.15}Zr_{0.1}Ti_{0.9}O_3$ ceramics. *Solid State Sci.* **52**, 10-18 (2016).
13. Tang, L. et al. Giant near-room-temperature pyroelectric figures-of-merit originating from unusual dielectric bistability of two-dimensional perovskite ferroelectric crystals. *Chem. Mater.* **34**, 8898-8904 (2022).

14. Navid, A.; Lynch, C. S.; Pilon, L. Purified and porous poly(vinylidene fluoride-trifluoroethylene) thin films for pyroelectric infrared sensing and energy harvesting. *Smart Mater. Struct*. **19**, 055006 (2010).

15. Dietze, M. et al. Thick film polymer-ceramic composites for pyroelectric applications. *J. Appl. Phys*. **101**, 054113 (2007).

16. Perea-López, N. et al. Photosensor device based on few-layered $WS_2$ films. *Adv. Funct. Mater*. **23**, 5511-5517 (2013).

17. Buscema, M. et al. Fast and broadband photoresponse of few-layer black phosphorus field-effect transistors. *Nano Letters* **14**, 3347-3352 (2014).

18. Yin, Z. et al. Single-layer $MoS_2$ phototransistors. *ACS Nano* **6**, 74-80 (2012).

19. Tao, Y. et al. Flexible photodetector from ultraviolet to near infrared based on a $SnS_2$ nanosheet microsphere film. *J. Mater. Chem. C* **3**, 1347-1353 (2015).

20. Liu, Y. et al. Plasmon resonance enhanced multicolour photodetection by graphene. *Nat. Commun*. **2**, 579 (2011).

21. Patel, M.; Kim, H-S.; Kim, J. Wafer-scale production of vertical SnS multilayers for high-performing photoelectric devices. *Nanoscale* **9**, 15804-15812 (2017).

22. Ma, N.; Zhang, K.; Yang, Y. Photovoltaic-pyroelectric coupled effect induced electricity for self-powered photodetector system. *Adv. Mater*. **29**, 1703694 (2017).

23. Chen, J, et al. Self-driven ultraviolet photodetectors based on ferroelectric depolarization field and interfacial potential. *Sensors Actuat. A-Phys*. **315**, 112267 (2020).

24. Sun, M. X. et al. Heterostructured graphene quantum dot/$WSe_2$/Si photodetector with suppressed dark current and improved detectivity. *Nano Res*. **11**, 3233-3243 (2018).

25. An, X. et al. Tunable graphene-silicon heterojunctions for ultrasensitive photodetection. *Nano Letters* **13**, 909-916 (2013).

26. Hong, Q. et al. Self-powered ultrafast broadband photodetector based on *p-n* heterojunctions of CuO/Si nanowire array. *ACS Appl. Mater. Interfaces* **6**, 20887-20894 (2014).

27. Zheng, L, et al. Large scale, highly efficient and self-powered UV photodetectors enabled by all-solid-state *n*-$TiO_2$ nanowell/*p*-NiO mesoporous nanosheet heterojunctions. *J. Mater. Chem. C* **4**, 10032-10039 (2016).
